# The effect of small solar powered ‘Bͻkͻͻ’ net fans on mosquito net use: results from a randomized controlled cross-over trial in southern Ghana

**DOI:** 10.1186/s12936-016-1654-2

**Published:** 2017-01-03

**Authors:** Olivier J. T. Briët, Joshua O. Yukich, Constanze Pfeiffer, William Miller, Mulako S. Jaeger, Nitin Khanna, Samuel Oppong, Peter Nardini, Collins K. Ahorlu, Joseph Keating

**Affiliations:** 1 Swiss Tropical and Public Health Institute, Basel, Switzerland; 2 University of Basel, Basel, Switzerland; 3 Department of Tropical Medicine, Tulane University School of Public Health and Tropical Medicine, New Orleans, USA; 4 Center for Applied Malaria Research and Evaluation, Tulane University, New Orleans, USA; 5 Green World Health Net, Albuquerque, USA; 6 National Malaria Control Programme, Accra, Ghana; 7 Noguchi Memorial Institute for Medical Research, University of Ghana, Accra, Ghana

**Keywords:** Insecticide treated net, Net use, Ghana, Solar power, Fan, Malaria, Hawthorne effect

## Abstract

**Background:**

Long-lasting insecticidal nets (LLINs) are ineffective malaria transmission prevention tools if they are unused. Discomfort due to heat is the most commonly reported reason for not using nets, but this problem is largely unaddressed. With increasing rural electrification and the dropping price of solar power, fans could improve comfort inside nets and be affordable to populations in malaria endemic areas. Here, results are presented from a pilot randomized controlled cross-over study testing the effect of fans on LLIN use.

**Methods:**

Eighty-three households from two rural communities in Greater Accra, Ghana, randomized into three groups, participated in a 10-month cross-over trial. After a screening survey to identify eligible households, all households received new LLINs. Bͻkͻͻ net fan systems (one fan per member) were given to households in Group 1 and water filters were given to households in Group 2. At mid-point, Group 1 and 2 crossed over interventions. Households in Group 1 and 2 participated in fortnightly surveys on households’ practices related to nets, fans and water filters, while households in Group 3 were surveyed only at screening, mid-point and study end. Entomological and weather data were collected throughout the study. Analysis took both ‘per protocol’ (PP) and ‘intention to treat’ (ITT) approaches. The mid- and end-point survey data from Group 1 and 2 were analysed using Firth logistic regressions. Fortnightly survey data from all groups were analysed using logistic regressions with random effects.

**Results:**

Provision of fans to households appeared to increase net use in this study. Although the increase in net use explained by fans was not significant in the primary analyses (ITT odds ratio 3.24, p > 0.01; PP odds ratio = 1.17, p > 0.01), it was significant in secondary PP analysis (odds ratio = 1.95, p < 0.01). Net use was high at screening and even higher after provision of new LLINs and with follow up. Fan use was 90–100% depending on the fortnightly visit.

**Conclusions:**

This pilot study could not provide definitive evidence that fans increase net use. A larger study with additional statistical power is needed to assess this association across communities with diverse environmental and socio-demographic characteristics.

**Electronic supplementary material:**

The online version of this article (doi:10.1186/s12936-016-1654-2) contains supplementary material, which is available to authorized users.

## Background

Long-lasting insecticidal nets (LLINs) and indoor residual spray are the two core interventions the World Health Organization (WHO) recommends for vector control. The WHO also recommends that areas with moderate to high malaria transmission achieve universal coverage with vector control, including ownership and use of LLINs [1]. Insecticide treated nets (ITNs), including LLINs, have been credited with the highest number of malaria cases averted since the year 2000 [2]. However, ITNs are ineffective if they are not being used. Population-based surveys show that net use among those owning an ITN varies widely among countries [3].

The literature cites several reasons for net non-use, including discomfort due to heat, social factors related to absence or disruption of sleeping arrangements and perceived low density of mosquitoes [4]. The inside of a mosquito net may feel stuffy during hot weather because the nets reduce ventilation [5], and it has been reported that mosquito net use varies depending on the level of nuisance biting mosquito presence and temperature [6].

Behaviour change communication (BCC) has the potential to improve net usage during warm weather and modifications to nets that increase the comfort level under the net could compliment educational or BCC interventions [4]. Mosquito net manufacturers can increase mesh size to an extent to allow ventilation, but are limited by mesh size specifications and perceived increased penetration by biting insects with larger mesh size [7].

After experimenting with prototypes of solar powered systems that included fans placed inside mosquito nets, dubbed the ‘Bͻkͻͻ System’, as ‘bͻkͻͻ’ (pronunciation follows the international phonetic alphabet) is Twi for ‘I am cool’ [8], it was hypothesized that such a low-energy solar net fan system could be desirable and economically affordable for a large part of the population in rural sub-Saharan African settings, and that access to such a fan system could increase bed net usage in areas with a hot climate [8, 9].

Here, results are presented of a small randomized controlled cross-over trial conducted in rural Ghana, testing whether provision of Bͻkͻͻ net fan systems increases individual net use.

## Methods

### Study setting

The trial was conducted in rural Greater Accra, an area with low (23.2%) net use despite 71% having access [10–12]. Of African countries surveyed with a demographic and health survey (DHS) in the 2012–2015 period, Ghana ranks near the bottom in net use, even among those reporting to have a net (57.5% use). Rural Greater Accra had the lowest use of all rural strata ([11, 12]). Southern Ghana has a humidity index (humidex) greater than 30 year round [9], indicating that this area is extremely hot and humid, which could be a barrier to mosquito net use as a result of discomfort. The humidex is an index first used by Canadian meteorologists combining temperature and dew points in a measure of how hot the weather feels. No LLIN mass distribution activities took place in the Greater Accra Region during 2013–2015. In the Greater Accra Region, the Dodowa Demographic Surveillance Site (DSS) in Shai-Osudoku District (formerly Dangme West District), specifically the villages of Apese (Abuminya) and Amanfro, were selected for this study. These villages were chosen because they lack access to the main electricity grid. Farming activities in the area include raising poultry, goats, pigs and cattle, and growing maize, cassava, mango and plantain.

### Study design

**Fig. 1:**
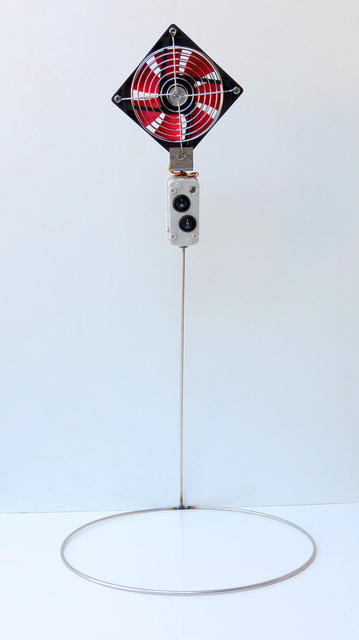
Bͻkͻͻ net fan

The randomized controlled cross-over trial, with households serving as the unit of randomization, followed a two-sequence, two-period, two-treatment design; additionally a third group was followed that received neither of the cross over interventions. The primary intervention was the Bͻkͻͻ net fan system, which consisted of a solar panel connected to a deep-cycle absorbed glass mat lead-acid battery via a charge controller, which was also connected to one or more net fans. A Bͻkͻͻ net fan is a 12 V, 0.8 W box-fan, with a switch for off, low (series circuit with a 60.4 Ω metal film resistor), and high setting, and a 0.1 W light emitting diode (LED) light with separate switch in the switch console, mounted on a stainless steel wire pedestal (Fig. 1). This pedestal was designed so as not to disturb the normal recommended hanging position of a net, allowing the net to be tucked-in and without risk of damage (Additional file 1). A water filter (Vestergaard LifeStraw^®^ Family 2.0) with capacity for the whole household was chosen as the alternative intervention and served as the control-intervention arm of the study. This household based alternative intervention was selected because it was not expected to have any effect on mosquito-net use.

During information workshops (one in each village) and house-to-house-visits, all 104 heads of households identified to (permanently) reside in two adjacent study villages were invited to participate in a screening survey designed to identify households eligible to participate in the study. During the screening survey, LLINs (TANA Netting DawaPlus^®^ 2.0) were distributed free of charge to participating households to cover all identified sleeping spaces, regardless of whether (other) nets were present. From the screening survey, 83 eligible households were identified, excluding 16 households with less than two members and the households of the two village chiefs and one health worker as well as two households that had members who assisted in doing installations of interventions. After consent to participate in the survey, households were randomly assigned into three intervention sequence groups: Group 1, which received fan systems (with one net fan for each household member) for the duration of the first period and water filters for the duration of the second period; Group 2, which received water filters for the duration of the first period and fan systems for the duration of the second period; and Group 3, which received neither intervention. The head of each household, or, in their absence, a person older than 15 years of age, in Groups 1 and 2 participated in fortnightly interviews, while household heads in Group 3 were only interviewed at screening, study mid- and end point. Fortnightly visits in Groups 1 and 2 were made to monitor correct technical functioning of the new solar power systems, and anticipated variability in weather and entomological conditions suggested frequent evaluation of fan and LLIN use throughout the study period. Prior to the study mid-point, qualitative in-depth key informant interviews with a selection of the participating households in Groups 1 and 2 were conducted, which are reported elsewhere [13]. Group 3 thus served as a control for possible effects of intense study contact through fortnightly surveys and in-depth interviews. After the mid-point survey at the end of the first study period, households in Groups 1 and 2 were crossed over. From households in Group 1, solar panels, battery and fans were removed, but electrical wiring and charge controllers were left installed to facilitate reinstallation at the end of the study and to further assure participants in Group 1 that solar power systems would be reinstalled. During the end-point survey (at the end of the second study period), all participating households in Groups 1, 2 and 3 received (ownership of) an installation of solar panels, battery and charge controller, as well as a water filter, and were given the opportunity to purchase as many net fans as there were household members through an individual level auction, results of which will be presented elsewhere (Yukich et al. pers. comm.).

### Measurement of outcomes and confounders

During each survey interview, the respondent was asked to report for all household members who spent the night at the household, whether they slept under a net, and if not, if a space under a net was available to them. Also, they were asked about fan use and switch setting and sleeping outside (part of) the night. In addition, other data such as education, age, occupation and health status were collected.

Three non-study households (those of the two village chiefs and the health worker), received fan systems and water filters as per the schedule of Group 1, but kept their solar power installation during cross-over in order to power Suna (BioGents) mosquito traps [14] hung on their verandas; these traps were emptied approximately three times per week, and the catch identified to genus level. The odour blend lures in the traps, MB5 (BioGents) [15], were refreshed once during the course of the study. No artificial source of carbon-dioxide or replacement was provided to attract mosquitoes. These data were used to understand variation in nuisance levels of nocturnal biting flying insects during the study period.

The temperature and humidity in same three households used for entomological data collection were monitored on an hourly basis throughout the study using EL USB 2+ high accuracy data loggers (Lascar) attached to bed frames. The 9 p.m. (indoor) temperature and relative humidity were used to calculate the humidex, an index used by Canadian meteorologists, to describe how hot the weather feels to the average person [16], which may affect net use behaviour.

### Sample size

Sample size calculations were done for the primary analysis plan, using only mid-point and end-point surveys in Groups 1 and 2, using the R package clusterPower [17]. Households were assumed to consist of four members all of whom can sleep under a mosquito net the night before any survey. The between-household variance was assumed to be 0.1 in both survey rounds. For each sample size scenario, 100 simulations were conducted. All simulations assumed that data would be analysed using logistic regression. It was also assumed that with the control-intervention (water filters), 80% of household residents would have used a net the night before the survey. The calculations indicated that approximately 25 households per arm were required to detect a 10 per-cent point increase in bed net usage from an 80% screening level with 80% power and a 5% alpha level.

### Data management

Survey data were collected and entered using password protected hand-held android tablet computers (Samsung Galaxy Tab 3 7.0 Lite 8 GB) with questionnaires programmed in open data kit (ODK) [18] with customized entry screens, and then sent to and stored on a secure server.

### Statistical analysis

Chi square and *t* test statistics were used to test for significant differences between randomization groups at screening. A p value of less than 0.05 was used to indicate a statistically significant difference in characteristics and outcomes. Empirically estimated standard errors were used to control for intra-class correlation within households at the bivariate level. Due to problems with model convergence as a result of low-sample sizes within the frequency tables, the primary statistical analysis used Firth logistic regression applied to mid- and end-point survey data from Groups 1 and 2. Firth logistic regression, also called penalized likelihood model, accounts for the effects of small cell sample sizes, and this technique is useful when the data are unbalanced and there is a highly significant predictive factor in the model [19]. For the primary analysis, missing outcome data was imputed using a last observation carried forward method, the last observation data coming from the most recent fortnightly survey preceding the missing outcome. All primary analyses were done using STATA v. 13 (Stata Corporation, College Station Texas). The secondary statistical analysis applied logistic regression with person-level random effects to fortnightly survey data from all groups, using the R software package ‘lme4’. The explanatory variable ‘intensity of follow up’, a binary variable with value one for fortnightly surveys (after the screening survey) in Groups 1 and 2, and zero for screening surveys and surveys in Group 3 (where households were only surveyed during screening, mid-point and end-point), was included to control for a possible effect of intense study contact (sometimes referred to as ‘Hawthorne effect’ [20]).

Primary and secondary analyses took both an ‘intention to treat’ (ITT) approach, with all households enrolled in the trial analysed according to their randomization group, and a ‘per protocol’ (PP) approach, which omitted data from households that received the wrong intervention.^1^

## Results

### Trial profile

Out of 104 screened households, 83 were eligible for inclusion in the study. Because only twenty-seven solar power systems were available for random allocation at the start of the study, 27 households were randomly allocated to Group 1, 30 households to Group 2 and the remaining 26 households were allocated to Group 3.

**Fig. 2:**
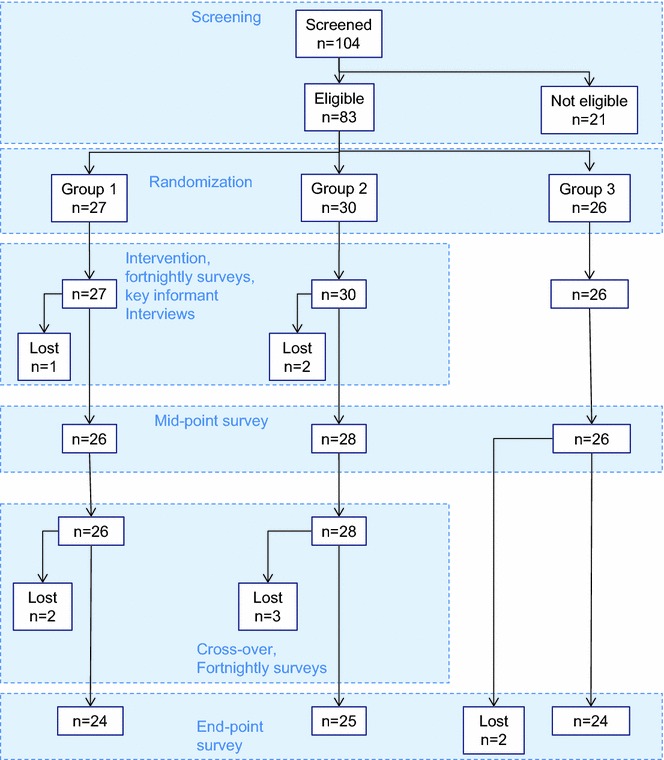
Study profile

The trial profile is illustrated in Fig. 2 (for details on interventions, see Additional file 2: Figure S1). During the course of the trial, there were six households that deviated from the trial schedule due to exchanges of interventions: during the first period, one household in Group 1 donated the fan system to a household in Group 3 (which from then on followed the schedule of Group 1); one household in Group 2 received a fan system during the first period and a water filter during the second study period; and two households in Group 3 received filters during the first period and a fan system during the second period; also, one household in Group 2 did not receive a fan system during the second period. Data from these six households were included in ITT analysis and treated according to their randomization group, but excluded from PP analysis. Three households were lost to follow up during the first period, and seven were lost to follow up during the second study period. No problems were encountered at cross-over with collecting fans and filters from participating households in Groups 1 and 2, respectively.

In the primary analysis, the following data was carried forward: for the mid-point survey, in Group 1, one household’s observations were carried forward from a fortnightly survey, and in Group 2, three households’ observations were carried forward from a fortnightly survey; for the end-point survey, in Group 1, two households’ observations were carried forward from a fortnightly survey, and in Group 2, three households’ observations were carried forward from a fortnightly survey. At mid-point, there was one household in Group 1 where no members had slept at home the previous night, but such missing data were not imputed by carrying forward earlier observations.

### Characteristics at screening

Table 1 presents characteristics of individuals included in the screening survey for Group 1 (i.e. those individuals in households to receive the net fan first), Group 2 (i.e. those individuals in households to receive the net fan during the second time-period after cross-over) and Group 3 (i.e. those individuals where data were collected during screening, mid-point and end-point surveys, but no introduction of intervention took place). Overall characteristics were similar between groups during screening, with the majority of individuals reporting farming as their principal form of employment in Group 1 (29.4%, 95% confidence interval [CI] = 19.0–42.5), Group 2 (31.1%, 95% CI = 24.7–38.4), and Group 3 (27.1%, 95% CI = 20.8–34.6). In addition, a majority of individuals had at least a primary school-level education in Group 1 (46.1%, 95% CI = 32.8–60.0), Group 2 (42.6%, 95% CI = 34.1–51.6), and Group 3 (51.7%, 95% CI = 42.8–60.5). No statistically significant differences between characteristics of Groups 1 and 2 individuals were detected during screening between occupation, sex, education, those reporting diarrhoea in the past two weeks, and those reporting a cough in the past two weeks. Statistically significant (p < 0.01) differences in net use were however detected, with Group 2 reporting higher net use at screening (60.7%, 95% CI = 41.9–76.7). However, amongst those having access to a net (defined as having slept under a net, or if not, having indicated that a space under a net was available), net use was very high at 91.3% (95% CI = 83.9–95.5), and there was no significant difference between groups.

**Table 1:** Descriptive statistics for individuals (all ages) in sampled houses during screening, by randomization group

	Group 1		Group 2		Group 3	
	(n = 102)		(n = 122)		(n = 118)	
	%	95% CI	%	95% CI	%	95% CI
Occupation						
No employment	7.8	3.9–15.3	4.1	1.6–10.3	7.6	3.6–15.4
Student	16.7	6.5–36.4	23.0	13.4–36.5	28.8	20.9–38.3
Farmer	29.4	19.0–42.5	31.1	24.7–38.4	27.1	20.8–34.6
Other occupation	21.6	13.3–33.1	18.0	12.2–25.8	17.8	11.7–26.1
Child (too young to work)	24.5	14.5–38.4	23.8	14.5–36.5	18.6	10.5–30.9
Sex						
Male	49.0	41.3–56.8	46.7	38.9–54.7	44.9	38.7–51.3
Female	51.0	43.2–58.7	53.3	45.3–61.1	55.1	48.7–61.3
Education						
No formal education	24.5	15.0–37.4	22.1	15.4–30.7	18.6	11.8–28.1
Primary	46.1	32.8–60.0	42.6	34.1–51.6	51.7	42.8–60.5
Secondary or higher	0.9	0.1–6.9	6.6	3.2–13.1	4.2	1.7–10.4
Other (Kindergarten or younger)	28.4	18.5–41.0	28.7	20.1–39.2	25.4	17.9–34.7
Health						
Fever in last 2 weeks	33.3	22.8–45.8	44.3	31.8–57.5	30.5	21.7–41.0
Cough in last 2 weeks	27.5	20.0–36.4	32.8	22.9–44.5	25.4	17.8–34.9
Diarrhoea in last 2 weeks	20.6	13.5–30.2	24.6	15.2–37.2	18.6	11.2–29.5
Mosquito net use						
Net used last night**	45.1	26.0–64.7	60.7	41.9–76.7	40.7	24.5–59.2

Standard errors adjusted for intra-class correlation at the household level

** p < 0.01

### Entomology

**Fig. 3:**
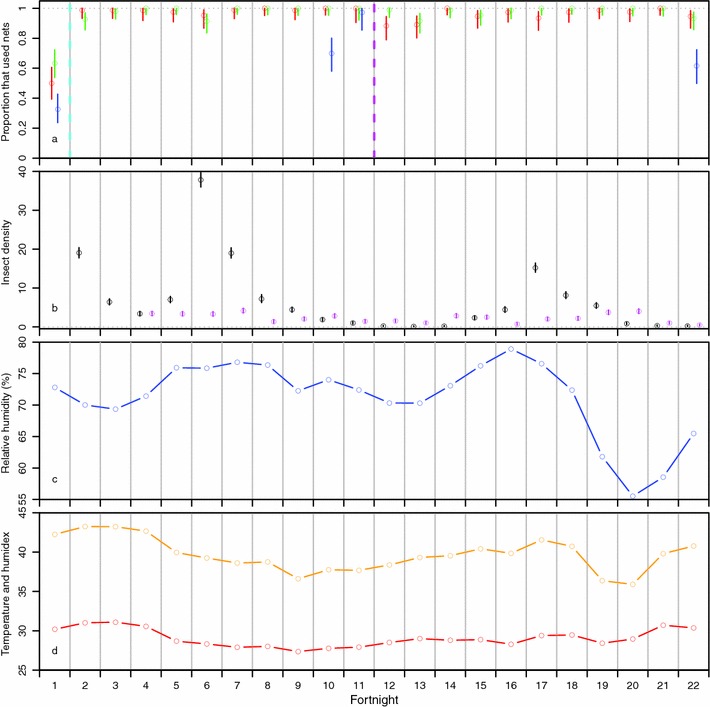
Fortnightly results in per protocol analysis. **a** Shows the proportion of people that used bed nets (irrespective of access) per fortnight, with Group 1 in *red*, Group 2 in *green*, and Group 3 in *blue*. *Vertical lines* show 95% Clopper-Pearson exact confidence intervals. The *cyan vertical dashed line* demarcates the start of the intervention, and the *magenta dashed line* demarcates the cross-over of interventions between Groups 1 and 2. **b** Shows the average nightly density of female mosquitoes (*black*) and female sand flies (*magenta*), per fortnight. *Vertical lines* show 95% exact Poisson confidence intervals. **c** Shows the average 9 p.m. relative humidity per fortnight. **d** Shows the average 9 p.m. temperature in degrees Celsius in *red* and humidex (unit less) in *orange* per fortnight

The mean number of female mosquitoes and female sand flies per trap per night is displayed in Fig. 3b (data aggregated per fortnight). Data for sand flies were not collected during the first three fortnights. Out of 5929 female mosquitoes (Diptera: Culicidae), 93.4% were *Anopheles*, 4.6% were *Culex*, 1.6% were *Mansonia* and 0.3% were *Aedes*. Also, 1837 female sand flies (Diptera: Psychodidae: Phlebotominae) were caught (27.4% of biting insects caught during the last 19 fortnights). There were fluctuations in the densities of biting flies, with densities being very low during September 2015 (fortnights 12 and 13, with on average 0.13 mosquitoes and 1.07 sand flies per trap per night) and January 2016 (fortnights 21 and 22, with on average 0.20 mosquitoes and 0.79 sand flies per trap per night). Details of entomological collections are given as Additional file 3.

### Indoor climate

Figure 3c shows the fortnightly average 9 p.m. temperature and humidex during the study period in the bedrooms of the two chiefs and health worker. The 9 p.m. temperature varied between 26 °C and 32.5 °C over the study period. In the period 15/12/2015–14/01/2016 (roughly, fortnights 19–21), the average 9 p.m. relative humidity was below 60% for 21 out of 31 days, resulting in relatively low humidex values. Relative humidity at 9 p.m. varied over the study period between 23.3 and 84.5% (Additional file 4).

### Mosquito net use in screening, mid-point and end-point surveys

The main outcome of the study was bed net use,^2^ among those who slept in a study household the night before the survey. Table 2 presents the proportion of individuals using nets the night before the survey by randomization group in ITT and PP analyses for screening, midline, and end-line data collection points. Across all groups, net use was 49.1% (95% CI = 38.2–60.1) during the screening survey.

**Table 2:** Mosquito net use the night before the survey in households at screening, midline, and endline data collection points, by group

Analysis	Group 1				Group 2				Group 3			
	N	n	%	95% CI	N	n	%	95% CI	N	n	%	95% CI
ITT												
Screen**	27	102	45.1	26.9–64.7	30	122	60.7	41.9–76.7	25	118	40.7	24.5–59.2
Mid**	25	88	94.3	69.6–99.2	28	85	100		25	128	78.9	65.4–88.1
End**	25	83	89.2	69.9–96.7	28	98	89.8	65.9–97.6	24	97	69.1	48.3–84.2
PP												
Screen**	25	92	50.0	30.7–69.3	27	112	63.4	43.1–79.9	22	101	32.7	17.7–52.3
Mid**	24	75	100		27	83	100		21	112	76.8	62.1–87.0
End**	24	78	94.9	86.7–98.1	27	92	93.5	74.9–98.6	21	84	64.3	42.4–81.5

Standard errors adjusted for intra-class correlation at the household level

*N* number of households, *n* number of individuals, *CI* confidence interval, *ITT* intention to treat, *PP* per protocol

** p < 0.01

Additional file 2: Table S1 shows p values of F-tests comparing paired observations. In ITT analysis, there were no significant differences between groups at different survey points, but in PP analysis, net use in Group 3 was significantly different (F-test, p < 0.05) at the end of the first study period and at the end of the second study period from net use in both Group 1 and Group 2, and Group 3 was also significantly different from Group 2 during screening. Between surveys, net use was significantly higher at the end of the first study period and at the end of the second study period compared to during screening, but not different between study period. The differences between Groups 1 and 2 at the end of the first study period and at the end of the second study period were not statistically significant.

Additional file 2: Table S2 shows net use conditional on access. Across all groups, net use conditional on access was 91.3% (95% CI = 83.9–95.5) during the screening survey.

### Mosquito net use using fortnightly survey data

Figure 3a shows fortnightly variations in mosquito net use during the study period in the three groups in PP analysis. Mosquito net use was much higher from fortnight 2 onwards than at screening (fortnight 1), after the distribution of free LLINs. Net use was slightly higher in Group 1 (with fans) compared to Group 2 during the first period (fortnights 2–11), while after cross-over, it was slightly higher in Group 2 (then with fans) compared to Group 1 during the second period (fortnights 12–22).

### Regression results

Table 3 presents results from the Firth logistic regressions for both ITT and PP primary analysis using mid-point and end-point survey data of Groups 1 and 2. This analysis lacked power to detect a significant association between having the fan system and increased net use [ITT odds ratio (OR) = 3.24, 95% CI = 0.70–14.92; PP OR = 1.17, 95% CI = 0.15–9.22]. Net use at the end-of the second study period was significantly lower across both groups compared to at the end of the first study period. Net use in Group 1 was not significantly different from that in Group 2.

**Table 3:** Firth logistic regression results analysing the effect of intervention on mosquito net use in intervention groups in mid-point and end-point surveys

LLIN use night before the survey	Intention to treat analysis (n = 354)		Per protocol analysis (n = 328)	
	OR	95% CI	OR	95% CI
Group membership				
Group 1	0.29	0.06–1.32	1.06	0.13–8.34
Group 2 (reference)	1.00		1.00	
Survey				
End of 2nd period	0.16*	0.03–0.74	0.09*	0.01–0.73
End of 1st period (reference)	1.00		1.00	
Intervention				
Received fans	3.24	0.70–14.92	1.17	0.15–9.22
Did not receive fans (reference)	1.00		1.00	
Constant	52.82**	11.46–243.42	142.37**	18.11–1119.01
Wald chi^2^	5.68		5.29	

Group 1 received fans during the first time period; group 2 received the fan console during the second time period (crossover)

* p < 0.05

** p < 0.01

Results of the person-level random effects regressions with net use as outcome are presented in Table 4. This regression uses data from all surveys and groups, except those from the screening survey, because at screening, net use was negatively affected by there not being enough nets for everyone in the community. In (Additional file 2: Figure S2), fortnightly results for the alternative outcome net use conditional on access (if the household member had had access to a sleeping place under a net) are shown, and, in (Additional file 2: Table S2), a regression with this outcome, using also the screening data, is presented. Results in Table 4 show that the effect of intensity of follow up was very strong and significant in both ITT (OR = 45.18, 95% CI = 28.17–75.30) and PP (OR = 39.88, 95% CI = 25.82–64.09) analyses. The positive effect of net fans on net use was significant in PP analysis (OR = 1.95, 95% CI = 1.21–3.21), but not in in ITT analysis (OR = 1.33, 95% CI = 0.87–2.05). The analysis of net use conditional on access showed similar results (Additional file 2).

**Table 4:** Logistic regressions with person-level random effects (with odds ratios presented) predicting effect of intervention on mosquito net use in study communities using data from all groups and (fortnightly) surveys except screening

	Intention to treat analysis		Per protocol analysis	
	(n = 471)		(n = 425)	
	OR	95% CI	OR	95% CI
Fans				
Net fans	1.33	0.87–2.05	1.95**	1.21–3.21
No fans (reference)	1		1	
Intensity of follow up				
Strong	45.18**	28.17–75.3	39.88**	25.82–64.09
Weak (reference)	1		1	
Constant	1.76**	1.31–2.37	1.42**	1.1–1.85

*OR* odds ratio, *CI* confidence interval

** p < 0.01

### Outdoor sleeping behaviour in fortnightly surveys

The proportion of people sleeping outdoors at least part of the night varied according to fortnight and group between 0 and 38.5% (Additional file 2: Figure S3) and was highest during fortnights 2 and 3. It was somewhat lower in household members that had access to fans, but the effect was not significantly different.

### Fan use in fortnightly surveys

The fortnightly use of fans by people with access to fans varied between 90 and 100% (Additional file 2: Figure S4). Most people used the fan on the high output setting (0.8 W) throughout the night, but also many people used the low setting or a combination of both high and low during the night. After cross-over (fortnight 12), the use of the low (and mixed) setting was initially much higher with Group 2 getting (used to) the fans as compared to Group 1 at the end of period 1. Use of the high setting by Group 2 gradually increased until fortnight 18, and then decreased.

## Discussion

While the direction of effect found in the study was consistent across all analyses of the data, the magnitude and statistical significance varied. Primary ITT and PP analyses had no power in this study to detect a significant effect of fans on LLIN use due to much higher LLIN use in study households than was expected. Increased levels of net use are likely attributable, at least partially, to a ‘Hawthorne effect’ resulting from intense fortnightly surveys applied in intervention groups as is evidenced by the much lower use rates reported in the third group which was not subjected to this follow up. Secondary analyses applied to fortnightly data and analyses of net use conditional on individual access to a sleeping space covered with an LLIN confirmed this increase in use when individuals had access to a net fan system.

Net use behaviour, measured as use among those having access, was (already) very high at screening of 91.3% in the study community, and this was much higher than anticipated based on analysis of 2014 DHS data, which indicated 57.5% use conditional on access in rural Greater Accra. Note that access in this study was defined as having slept under a net, or if not, having indicated that a space under a net was available, whereas in the reanalysis of the DHS data, access was defined as having slept under a net, or if not, if a space under a net was available, assuming two free spaces for an unused net and one space for a net that was already occupied by another person [10]. Intense study contact, in the form of fortnightly visits made by study personnel to participating intervention households, positively influenced net use: members of Group 3 (those not receiving either intervention or fortnightly visits) had lower net use than Groups 1 and 2. In future studies of fan interventions, a concerted effort should be made to minimize influencing study participants’ behaviour through study personnel contact. This contamination may have altered the estimated effect size of the fan systems in ITT analysis, though this would not be expected in PP analysis.

## Conclusions

Within the context of rural communities in Ghana without access to the electricity grid and with year-round malaria transmission, this pilot study could not provide definitive evidence that the net fan system increases net use. However, the increased odds of net use when having the net fan system is highly suggestive of such a relationship, which is further supported by a significant effect found in secondary PP analysis on fortnightly observations. Given the exceptionally high net use, partly associated with fortnightly study contact, and observed contamination due to non-adherence to the trial schedule, a larger study with additional statistical power is needed to assess the association between net use and net fan use across communities with diverse environmental and socio-demographic characteristics.

## Additional files

**Additional file 1.** Description of the Bɔkɔɔ net fan console development history.

**Additional file 2.** Additional table and figures.

**Additional file 3.** Entomological data.

**Additional file 4.** Weather (indoor temperature and humidity) data.

## Abbreviations

BCC: behaviour change communication
CI: confidence interval
DHS: demographic and health survey
DSS: demographic surveillance site
GWHN: Green World Health Net
ITN: insecticide treated net
ITT: intention to treat
LED: light emitting diode
LLIN: long lasting insecticidal net
NMIMR: Noguchi Memorial Institute for Medical Research
ODK: open data kit
OR: odds ratio
PP: per protocol
Swiss TPH: Swiss Tropical and Public Health Institute
WHO: World Health Organization

## Footnotes

1 The interventions were considered valuable by trial staff and participants, and when fan systems or water filters became available as some participating households moved out of the area, these fans or water filters were re-distributed to other households resulting in some contamination between study arms.

2 During a meeting to inform study villages and participants of study results, the chief of one village mentioned that without a fan during hot nights, nets are often used with the side panels left open to air out the net, only to be closed if mosquitoes are present. In this study, such type of ‘net use’ was not distinguishable from ‘correct net use’ with the sides closed at all times during the night.
