# Supplementary material for: The effect of small solar powered ‘Bͻkͻͻ’ net fans on mosquito net use: results from a randomized controlled cross-over trial in southern Ghana
Source: Malar J. 2017 Jan 3;16:12. doi: 10.1186/s12936-016-1654-2 (PMC5209841; doi:10.1186/s12936-016-1654-2)
Supplement: Supplementary file 1 — Additional file 1. Description of the Bɔkɔɔ net fan console development history. [file 12936_2016_1654_MOESM1_ESM.pdf]

Additional file 1 Description of the Bɔkɔɔ net fan console development history

The Bɔkɔɔ net fan console (Figure A1) is a second generation prototype (herein referred to as Prototype II) that was utilized for the randomized controlled cross-over trial in southern Ghana. Prototype II is an evolution from an initial prototype (herein referred to as Prototype I) developed by Peter Nardini in 2008 and tested in Ghana in 2012 [1,2].

Prototype I was put together using a criterion that at least ninety per cent of Bɔkɔɔ Fan parts should be available locally in small towns that have hardware and electronics shops in low and middle income countries. This criterion was relaxed in Prototype II to allow for improvements and modifications deemed important to increase the effectiveness and desirability of the Bɔkɔɔ Fan console and to get closer to a marketable product. There has also been considerable improvement in muffin fan efficiency since 2008 when Prototype I was first constructed. There remain opportunities for people to adapt the Prototype I design for DIY (do-it-yourself) construction which could enhance the distribution of the Bɔkɔɔ Fan and create alternative markets for this product.

Design work for Prototype II was led by Olivier J.T. Briët and Hans Kleiber with input from William Miller and Peter Nardini. Modifications in relation to overall design and specifications were discussed and implemented. The resulting modifications to the Bɔkɔɔ Fan include:

1) Change of the stand design so that the Bɔkɔɔ net fan console can be wedged between the floor and a mattress and is contained entirely inside a LLIN, allowing the net to be tucked-in underneath the foot of the stand. Prototype I required a hole in net (that was sealed with a zipper), which was deemed to compromise the durability of the LLIN. The modified stand is also lighter, allowing for the ability to adjust placement of the console, as well as resistant to corrosion (stainless steel instead of steel).

2) Changes to the muffin fan to decrease speed, increase power efficiency, and decrease noise level. It was concluded that lower air flow was sufficient for the Prototype II fan based on unpublished research conducted by Olivier Briët [3]. The air flow in the Prototype I is 59 cubic feet per minute (CFM) whereas the airflow from the fan used in Prototype II is 44 CFM at the highest setting. The lower air flow (accompanied by a lower fan speed), as well as, have resulted in substantial increase efficiency with reduced power use from 3.8 watts to 1 watt. Additionally, a reduction in air flow and fan speed decreased the noise level from 37 dBA to 26 dBA, putting it under 30 dBA, the upper limit for bedrooms [4].

3) Prototype II has two variable fan speeds. Prototype I has one fan speed.

4) Switches and wires in Prototype II are encased in a non-conductive, water repellent plastic enclosure that houses the variable fan speed and light switches, reducing wire connection exposure, and thus reducing damage due contact with water and corrosion.

5) Prototype II has no sharp edges that a net could get hooked on with risk of ripping.

Prototype development is on-going and further ideas and modifications are being explored based on anecdotal and qualitative feedback from study participants as well as based on observations made by the research team. Plans for future development include continued and more structured end-user participation in the design process through participatory design and co-design activities and exercises [5]. Additionally, product engineers will be consulted to help in the creation of a marketable product that is low cost, durable and effective.

Prototype II has the following components:

- Stainless chromium steel 1.4301U-piece 30/28/30x30mm, custom made
- Stainless steel wire frame, custom made from A2 steel 4mm thickness
- Rocker switch, round, 1x ON - OFF - ON (SCI parts number WS R13-112 DAAA)
- Rocker switch, round, 1x ON -OFF (SCI parts number WS R13-112 AAAA)
- G4 Halogen lamp holder (Goobay®)
- G4 036 WS 12 V LED lamp (Goobay®)
- Xilence red wing 120 fan
- LK Phobya Y-cable 3Pin Molex to 3 pin Molex
- IP 54 Mini 75 mm x 37 mm x 40 mm thermoplast juntion box (Spelsberg)
- 60.4 Ohm metal film resistor
- STV-R butt connectors (4)
- Central installation hollow plug socket
- DC power cord with 2.1 mm plug 2.5 A Si, 2.0m
- 120x120mm protection grills (2 pieces)
- Stainless steel M4 bolt (40mm) and M4 nut

References:

1. GreenWorldHealthNet. Genesis of the Boko Bed Net System [Internet]. First published in 2010 under African Health Net. Edited and republished under Green World Health Net in 2013. Available from: https://www.youtube.com/watch?v=NLOTwRPB_n0
2. Hughes G. BOKO Bed Net Project [Internet]. First published in 2012 under African Health Net. Edited and republished under Green World Health Net in 2014 [cited 2016 Jun 14]. Available from: https://www.youtube.com/watch?v=bCcXe4_CHR3.
3. Short report: At a quarter Watt, a net fan can compensate for the loss of air velocity over a resting person due to hanging a bed net, Olivier J.T. Briët, 2012, unpublished.
4. Acceptable Noise- dBA- Levels, EngineeringToolBox.com, referenced 2010, http://www.engineeringtoolbox.com/decibel-dba-levels-d_728.html
5. Consumer Co-creation in New Product Development, Hoyer, W.D., Chandy, R., Dorotic, M., Kraft, and Singh, S.S., Journal of Service Research, 2010, 13(3): 283-296

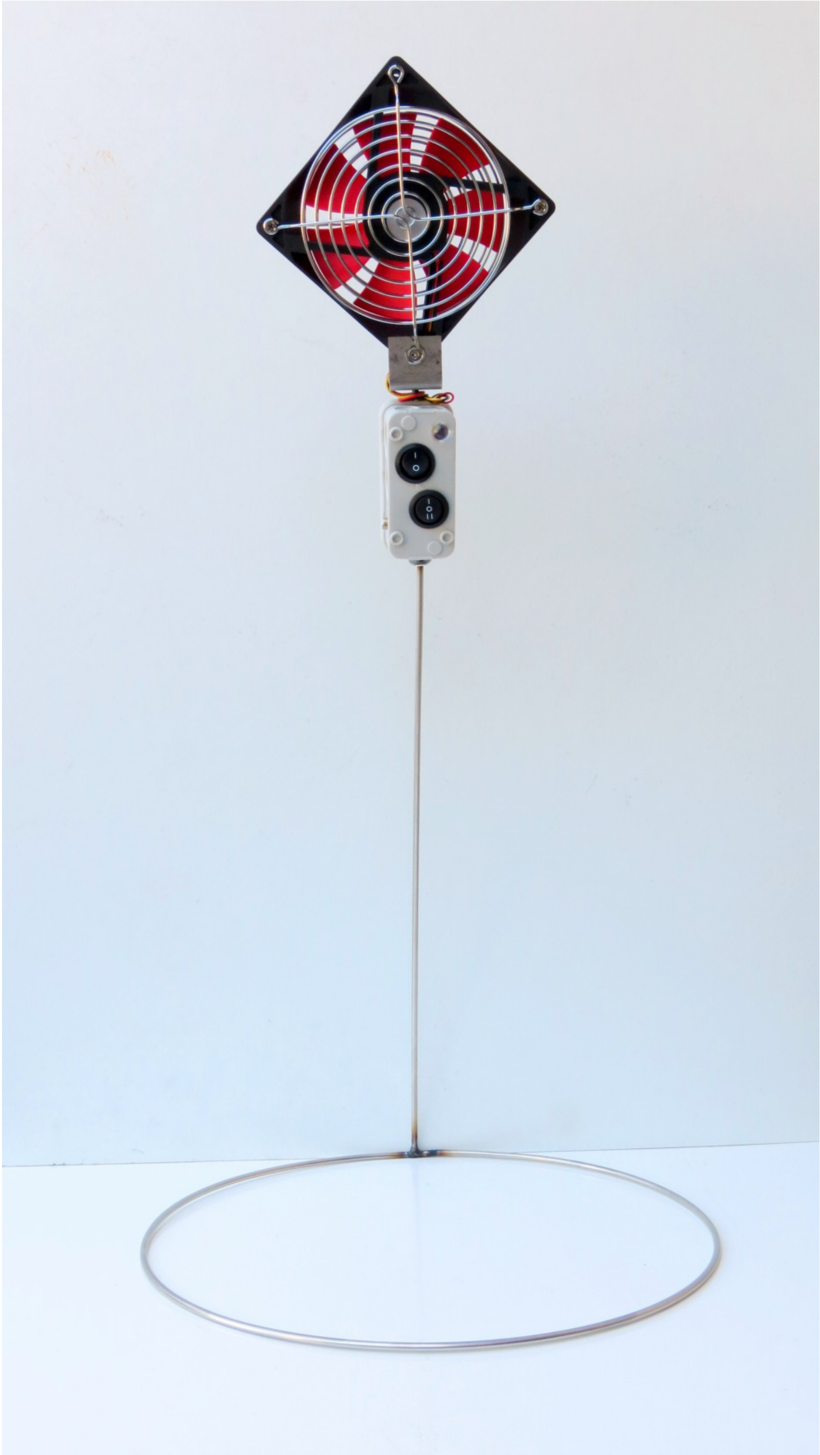

Figure A1 Bɔkɔɔ net fan (Prototype II)
