# Supplementary material for: The effect of small solar powered ‘Bͻkͻͻ’ net fans on mosquito net use: results from a randomized controlled cross-over trial in southern Ghana
Source: Malar J. 2017 Jan 3;16:12. doi: 10.1186/s12936-016-1654-2 (PMC5209841; doi:10.1186/s12936-016-1654-2)
Supplement: Supplementary file 2 — Additional file 2. Additional table and figures. [file 12936_2016_1654_MOESM2_ESM.pdf]

Additional file 2 Additional table and figures

Table S1A: Results from pair-wise comparisons (F-tests) of net use between groups at screening, mid-point and end-point surveys, with p-values presented.

	Survey		
	screening	mid	end
ITT			
G1 vs G2	0.2612	0.2917	0.9458
G1 vs G3	0.7456	0.1375	0.0855
G2 vs G3	0.1358	0.3638	0.1097
PP			
G1 vs G2	0.3502	NA (NS)	0.7884
G1 vs G3	0.2166	0.0052	0.0003
G2 vs G3	0.0318	0.0036	0.0146

G1: Group 1; G2: Group 2; G3: Group 3; NA: Not available; NS: Not significant; vs: versus; p-values <0.05 are highlighted in green.

Table S1B: Results from pair-wise comparisons (F-tests) of net use between surveys in Group 1, Group 2 and Group 3, with p-values presented.

	Group		
	1	2	3
ITT			
S vs M	0.0002	0.0002	0
S vs E	0.0001	0.0383	0.0036
M vs E	0.1438	0.1735	0.2768
PP			
S vs M	0.0001	0.0009	0
S vs E	0	0.014	0.0028
M vs E	0.0523	0.2041	0.2136

S: screening survey; M: mid-point survey; E: end survey; vs: versus; p-values <0.05 are highlighted in green.

Table S2: Mosquito net use conditional on having access to a net the night before the survey in households at screening, midline, and endline data collection points, by group.

Analysis	Group1				Group2				Group3			
	N	n	%	95%CI	N	n	%	95%CI	N	n	%	95%CI
**ITT**												
Screen**	16	51	90.2	71.7–97.1	21	80	92.5	78.4–97.7	16	53	90.6	76.7–96.5
Mid**	25	87	95.4	74.1–99.3	28	85	100		24	121	83.5	70.9–91.3
End**	24	77	96.1	88.1–98.8	28	98	89.8	65.9–97.6	19	75	89.3	73.4–96.2
**PP**												
Screen**	16	51	90.2	71.7–97.1	19	77	92.2	77.6–97.6	13	38	86.8	69.8–95.0
Mid**	24	75	100		27	83	100		20	105	81.9	68.0–90.6
End**	24	77	96.1	88.1–98.8	27	92	93.5	74.9–98.6	16	62	87.1	68.8–95.4

** P < 0.01; Standard errors adjusted for intra-class correlation at the household level; N = number of households; n = number of individuals; CI = confidence interval; ITT = intention to treat; PP = per protocol.

Table S3: Logistic regressions with person-level random effects predicting effect of intervention on mosquito net use conditional on access in study communities, using data from all groups and (fortnightly) surveys.

	**Intention to treat analysis** (n=435)		**Per protocol analysis** (n=394)	
	OR	95% CI	OR	95% CI
**Intervention**				
Bɔkɔɔ net fans	1.08	0.58–2.01	2.41*	1.16–5.35
No fans (reference)	1		1	
**Intensity of follow up**				
Strong	10.91**	4.4–27.6	16.28**	6.35–44.31
Weak (reference)	1		1	
**Constant**	1302.97**	346.3–5349.61	787.69**	187.59–3458.56

* P < 0.05; ** P < 0.01; OR: odds ratio; CI: confidence interval

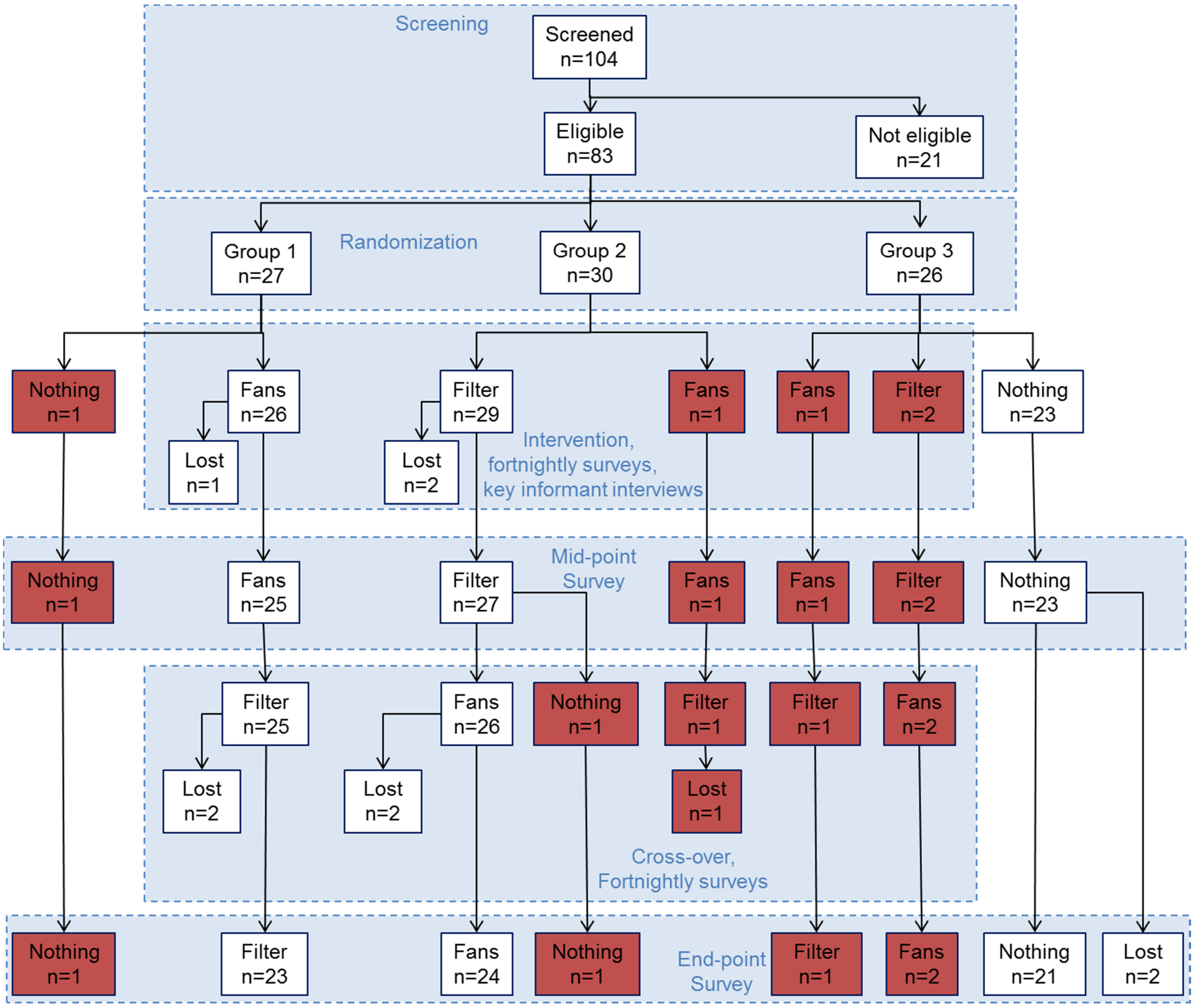

Figure S1: Study profile with intervention details. Data from households in red boxes were dropped from per-protocol analysis. Households that received neither fans nor filters are marked as 'Nothing'.

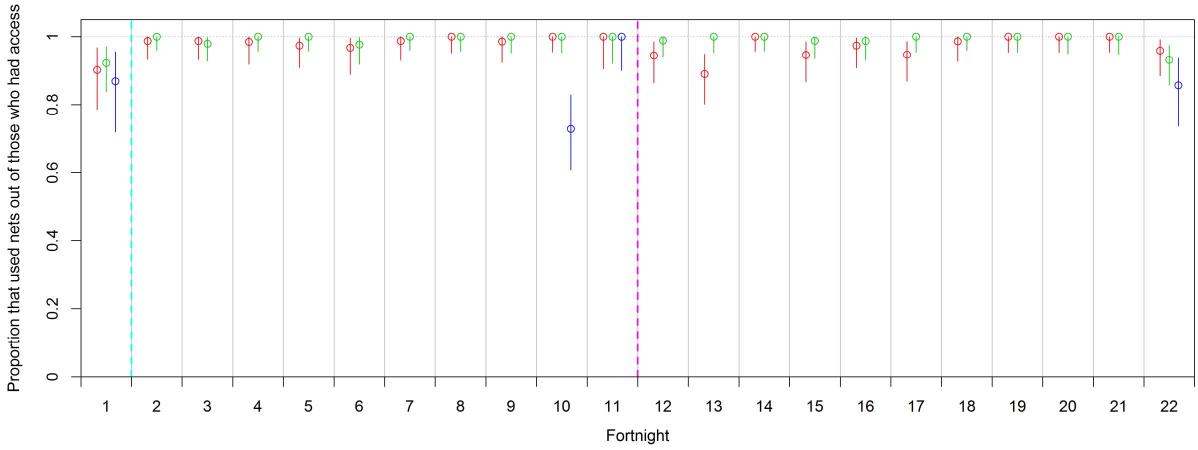

Figure S2 Fortnightly net use conditional on access. This figure shows the proportion of people that used bed nets out of those that had access in per protocol analysis per fortnight, with Group 1 in red, Group 2 in green, and Group 3 in blue. Vertical lines show 95% Clopper-Pearson exact confidence intervals. The cyan vertical dashed line demarcates the start of the intervention, and the magenta dashed line demarcates the cross-over of interventions between Group 1 and Group 2.

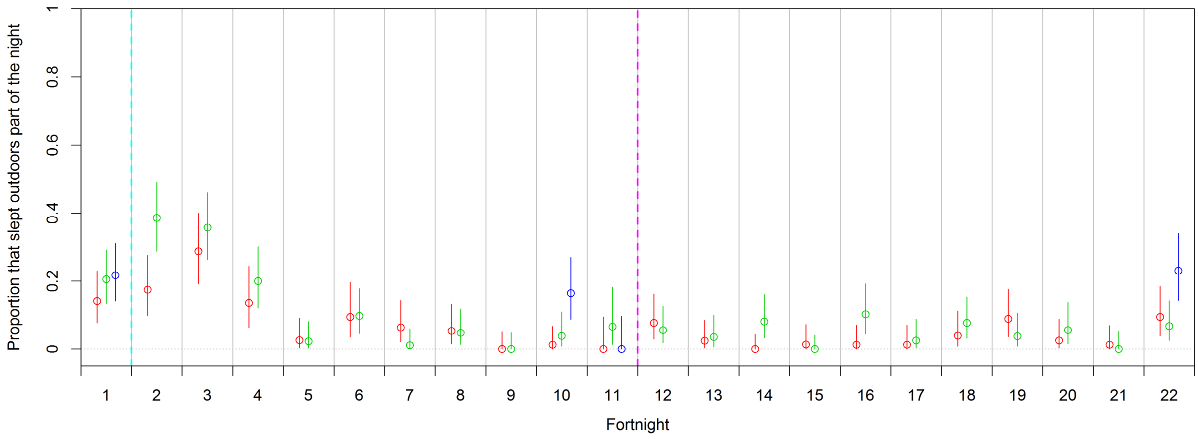

Figure S3 Fortnightly outdoor sleeping behaviour. This figure shows the proportion of people that slept outdoors at least part of the night in per protocol analysis per fortnight, with Group 1 in red, Group 2 in green, and Group 3 in blue. Vertical lines show 95% Clopper-Pearson exact confidence intervals. The cyan vertical dashed line demarcates the start of the intervention, and the magenta dashed line demarcates the cross-over of interventions between Group 1 and Group 2.

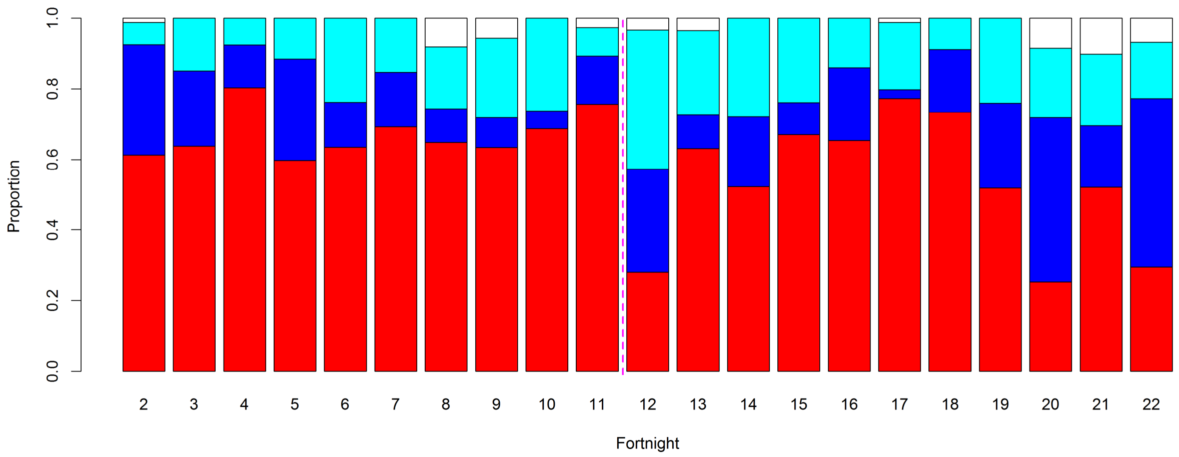

Figure S4 Fortnightly fan use. This figure shows the proportion of people that used the fan out of those that had access to a fan, and specifies the fan setting: high (red), mixed (dark blue), low (cyan), and not used (white). The magenta dashed line demarcates the cross-over of interventions between Group 1 and Group 2.
